# Health Equity of Rural Residents in Southwest China

**DOI:** 10.3389/fpubh.2021.611583

**Published:** 2021-03-23

**Authors:** Xiao-Mei Li, Jing Kou, Zhen Yu, Yuan-Yuan Xiao, Qiong Meng, Li-Ping He

**Affiliations:** ^1^School of Public Health, Kunming Medical University, Kunming, China; ^2^First Affiliated Hospital of Kunming Medical University, Kunming, China

**Keywords:** health equity, rural residents, Chinese healthcare reforms, disadvantaged area, household study

## Abstract

The Chinese government stresses healthcare reform to improve the health of all residents in urban and rural areas. However, much research showed that inequities still existed in health status and health services utilization in China, especially in economically disadvantaged areas. Southwest China's Yunnan Province is an ethnic frontier region with lagging economic development. This study analyzed health equity among rural residents with various socio-economic and demographic statuses in Yunnan Province. Research on this area concerns rural residents. Our study was based on a household study sample consisting of 27,395 participants from six counties in Yunnan. For all participants, data on demographic and socio-economic characteristics, and health status were collected. The chi-square test and logistic regression were used to analyze factors influencing health. The concentration index was used to evaluate health equity. For all respondents, the 2-week prevalence, the prevalence of chronic diseases, and the required hospitalization rate were 7.3, 12.8, and 9.2%, respectively. After adjusting the age proportion of the sixth population census of Yunnan Province, the 2-week prevalence was 7.1%, the prevalence of chronic disease was 10.7%, and the hospitalization rate was 8.4%. The concentration indexes (CIs) reflecting health equity among the respondents with different incomes and educational levels were negative. There was health inequity among respondents with different incomes and educational levels. The respondents with lower incomes and educational levels had worse health. The common influencing factors included gender, age, ethnicity, occupation, marriage status, and the number of family members. Females, the aged, ethnic minorities, farmers, and the divorced or widowed had worse health status than the control groups. Larger numbers of family members correlated with better health. The respondents with lower incomes or educational levels had higher chronic disease prevalences. The associations between the 2-week prevalence, required hospitalization rate, and age were U-shaped; the lowest age group and the highest age group had higher rates. In conclusion, more attention should be paid to females, the aged, ethnic minorities, farmers, the divorced or widowed, residents with low income and low educational level, and those with chronic diseases.

## Introduction

The term “health equity” has been defined by many researchers in the public health area, nevertheless, there is little consensus about its meaning (1). This lack of consensus is the principle that, motivates the elimination of disparities in health among various socioeconomic groups (2). The WHO/SIDA suggested that equity is different from equality; the former refers to the distribution of opportunities for survival that should be oriented toward individual needs (3). Pursuing health equity means “striving for equal opportunities for all social groups to be as healthy as possible, with selective focus on improving conditions for those who have had fewer opportunities” (1). Improving health is the ultimate goal of healthcare reform worldwide. The main target of the “Healthy China 2030” initiative proposed by the State Council of China is to achieve a higher national health level (4). To improve people's health, it is necessary to understand health status, identify factors influencing health, and study health equity.

The 4th and 5th National Health Services Survey (NHSS) of China in 2008 and 2013, respectively, found that the 2-week prevalences were 18.9 and 24.1%, respectively; the prevalences of chronic diseases were 24.1 and 33.1%, respectively; required hospitalization rates after medical diagnosis were 6.8 and 9.0%, respectively (5, 6). In other words, the demand for health care services increased in China, and the prevalence of chronic diseases increased rapidly. The 5th NHSS also reported that the required hospitalization rate after medical diagnosis for rural residents in western China was 9.4%, higher than the national average (9.0%).

Health service utilization experienced significant improvement in China since the establishment of China's New Rural Cooperative Medical System (NCMS) in 2003 (7). Healthy China 2020 issued by China's Ministry of Health in 2008, pointed out that citizens' health equity should be taken as an essential indicator to measure social justice and equity, and China's health reform and development should focus on eliminating inequities in health (8). Nevertheless, inequity still exists in health status and health services utilization, especially in economically disadvantaged areas (9–11). Nationwide health inequality exists in several areas, health status in eastern China was better than in other parts (12).

Yunnan is an ethnic frontier underdeveloped province located in southwest China with a minority population accounting for 33.34% of the total population. This study aims to analyze health equity for rural residents in Yunnan and explore the factors influencing rural residents' health equity in southwest China.

## Methods

### Participants

This study was based on a household survey from February to August 2013 with a cross-sectional design from Yunnan's six counties. The multi-stage sampling method was used to determine sample sources according to the regional economic level. First, the Dali Bai Autonomous Prefecture (a more developed region) and Zhaotong City (a less developed region) were selected out of 16 cities (regions). Second, three counties among 12 counties in Dali and three counties among 11 counties in Zhaotong were selected according to the economic levels (high, medium, and low). Third, two townships with different economic levels were randomly selected in each county. Fourth, the cluster sampling method was used to select one or two natural villages from each selected township. The household survey was conducted as face-to-face interviews with pretested structured questionnaires among all households in the selected villages.

The survey returned data on the 2-week prevalence, the prevalence of chronic disease, and the required hospitalization rate. Taking the results of the 5th NHSS as a reference, in which the required hospitalization rate (P) was 9.0%, α was 0.05, δ was 10%, the calculation of the sample size was as follows:

$$\begin{array}{l} n=\frac{{\left(z_{\alpha /2}\right)}^{2}∙\left(1-P\right)}{\delta^{2}∙P} \end{array}$$

The minimum sample size to be investigated in each region was 3,885. Because this was a cluster sampling, based on the design effect of two, the sample size was 7,770. Based on the average family population of four, 1942 households were investigated.

### Questionnaire

The questionnaire included two sections: (1) critical demographic and socioeconomic variables: age, gender, ethnicity, marital status, occupation, education level, annual household income, annual household expenditure, and family size; (2) health status: health condition over the past 2 weeks; required hospitalization over the past year; and chronic diseases over the past year. The reliability and validity of the questionnaire were evaluated using a pre-survey with a sample size of 120. Cronbach's alpha coefficient of 0.82 showed a good inner-reliability. The expert evaluation method was used to evaluate the content validity. The conclusion was that the items of the questionnaire reflected the research content. The questionnaire had ideal reliability and validity.

### Variables and Definitions

All respondents were divided into five age groups: 0–12, 13–20, 21–40, 41–60, and 61 years and above. The respondents were divided into five groups according to the quintiles of per capita income: very-low-income, low-income, middle-income, high-income, and very-high-income. Health status was comprehensively measured using the following three indexes: 2-week prevalence, required hospitalization rate, and prevalence of chronic diseases. The 2-week prevalence was calculated as the percentage of respondents who presented with or declared general malaise with or without treatment over the past 2 weeks. The 2-week prevalence refers to (1) conscious physical complaints, injury, or poisoning in the past 2 weeks with treatment measures (including self-treatment); (2) Physical complaints without any treatment measures, but for a few days' rest or stay in bed. The required hospitalization rate was calculated as the percentage of respondents who have required hospitalization after diagnosis by doctors over the past year. The “required hospitalization rate” is used instead of “hospitalization rate,” because although some residents are required to be hospitalized by health workers in local clinics, they do not go to a hospital for various reasons, such as economic reasons, inconvenient transportation, etc. The prevalence of chronic diseases was calculated as the percentage of respondents who had chronic diseases, such as hypertension and diabetes over the past year. Chronic disease refers to (1) A chronic disease (such as hypertension, diabetes, etc.) is diagnosed by a doctor in the past 1 year; (2) A chronic disease is diagnosed a year ago, and there are attacks within a year, and treatment measures were taken.

### Data Analysis

SPSS 18.0 (IBM Corporation, Armonk, USA) was used for statistical analysis. The chi-square test and univariate and multivariate logistic regression were used to analyze the influencing factors of health. In logistic regression, we used the ENTER method. All the independent variables have been included in the analysis, and the corresponding OR value of each variable is obtained by adjusting the influence of other independent variables. Concentration index (CI) and concentration curve based on a geometric approach were used to calculate health equity.

$$\begin{array}{l} G=1-\overset{n-1}{\underset{i=0}{\sum }}\left(x_{i+1}-x_{i}\right)\left(y_{i+1}+y_{i}\right) \end{array}$$

where *x*_*i*_ represents the cumulative percentage of the population ranked by income (education level) and *y*_*i*_ represents the cumulative percentage of the corresponding unhealthy population.

A two-tailed *P*-value ≤ 0.05 was considered statistically significant.

## Results

A total of 27,395 residents in 7,399 households were sampled from Dali Bai Autonomous Prefecture and Zhaotong City. All participants responded to the questionnaire. A total of 13,715 residents in 3,702 households were sampled from Dali, of which 5,316 residents in 1,256 households were in Dali City, 4,358 residents in 1,236 households in Weishan Yi and Hui Autonomous County, and 4,041 residents in 1,210 households in Midu County. There were 13,680 residents in 3,697 households sampled from Zhaotong City, of which 4,380 residents in 1,176 households were in Yanjin County, 4,404 residents in 1,258 households in Zhaoyang District, and 4,896 residents in 1,263 households in Ludian County.

### Health Status of the Respondents by Socio-Economic and Demographic Characteristics

Table 1 shows health status of the respondents. Approximately 51.8% of respondents were male. Most (83.9%) respondents were of Han ethnicity (the ethnic majority in China). The Bai ethnic group (12.5%) was the Dali's largest minority group; 59.8% were married, 64.8% were illiterate or only had a primary school education, 62.8% were farmers, and 13.8% were aged 61 years and above. Most of the respondents (98.8%) participated in China's basic medical insurance.

**Table 1:** Health status of the respondents [*n* (%)].

**Variables**	**Two-week prevalence**		**Prevalence of chronic diseases**		**Required hospitalization rate**	
	**%**	**95%CI**	**%**	**95%CI**	**%**	**95%CI**
Total	7.3	7.0, 7.6	12.8	12.4, 13.2	9.2	8.9, 9.5
**Age (years)**						
0–12	8.9***	8.1, 9.7	0.9***	0.6, 1.2	4.8***	4.2, 5.4
13–20	3.6	3.0, 4.2	1.1	0.8, 1.4	3.6	3.0, 4.2
21–40	5.0	4.5, 5.5	5.4	4.9, 5.9	6.7	6.2, 7.2
41–60	9.3	8.6, 10.0	21.0	20.1, 21.9	11.7	11.0, 12.4
61+	9.9	8.9, 10.9	37.0	35.5, 38.5	19.7	18.4, 21.0
**Marriage**						
Unmarried	6.1***	5.6, 6.6	1.5***	1.3, 1.7	4.3***	3.9, 4.7
Married	7.8	7.4, 8.2	17.6	17.0, 18.2	11.2	10.7, 11.7
Divorce/widowed	10.1	8.5, 11.7	34.0	31.5, 36.5	18.6	16.5, 20.7
**Average annual income (yuan)**						
<2,400	8.1	7.4, 8.8	18.4***	17.4, 19.4	10.5***	9.7, 11.3
2,400–4,399	7.4	6.7, 8.1	13.2	12.3, 14.1	8.6	7.8, 9.4
4,400–6,699	7.2	6.5, 7.9	11.5	10.6, 12.4	8.7	7.9, 9.5
6,700–10,199	6.9	6.2, 7.6	10.6	9.8, 11.4	9.3	8.5, 10.1
10,200+	7.5	6.8, 8.2	9.7	8.9, 10.5	8.2	7.5, 8.9
**Gender**						
Male	6.6***	6.2, 7.0	10.6***	10.1, 11.1	8.0***	7.6, 8.4
Female	8.0	7.5, 8.5	15.2	14.6, 15.8	10.4	9.9, 10.9
**Ethnicity**						
Han	7.1	6.8, 7.4	13.0*	12.6, 13.4	8.8***	8.4, 9.2
Bai	8.3	7.4, 9.2	12.0	10.9, 13.1	11.1	10.0, 12.2
Other	7.9	6.3, 9.5	10.4	8.6,2.2	10.3	8.5, 12.1
**Education**						
Illiterate	9.3***	8.6, 10.0	20.7***	19.8, 21.6	12.1***	11.4, 12.8
Primary school	6.9	6.4, 7.4	11.3	10.7, 11.9	8.9	8.3, 9.5
Middle school	6.1	5.5, 6.7	7.9	7.3, 8.5	7.1	6.5, 7.7
High school	6.0	4.7, 7.3	9.1	7.6, 10.6	7.9	6.5, 9.3
Above high school	7.1	5.7, 8.5	8.6	7.0, 10.2	7.2	5.7, 8.7
**Occupation**						
Farmers	8.1***	7.7, 8.5	17.7***	17.1, 18.3	11.5***	11.0, 12.0
Students	6.7	6.1, 7.3	1.1	0.8, 1.4	3.9	3.4, 4.4
Workers	2.5	1.7, 3.3	6.1	4.8, 7.4	4.8	3.7, 5.9
unemployed	6.7	4.8, 8.6	21.2	18.2, 24.2	14.3	11.7, 16.9
Others	5.7	4.5, 6.9	10.2	8.7, 11.7	7.7	6.3, 9.1

Chi-square test; *P < 0.05; **P < 0.01; ***P < 0.001.

The 2-week prevalence was 7.3%. It was highest in the youngest and oldest respondents whereas the respondents aged 13–20 years had the lowest 2-week prevalence. The divorced or widowed respondents had the highest rates, while the single respondents had the lowest rate. The illiterate residents had a higher rate than other educational level groups. Females had a higher rate than males. Concerning occupational distribution, workers had the lowest rate, and farmers had the highest rate. The most common diseases were influenza, headache, dizziness, diarrhea, and fever.

The prevalence of chronic diseases was 12.8%. Older age correlated with a higher prevalence of chronic diseases. The divorced or widowed respondents had the highest rates, and the single respondents had the lowest rate. Lower income correlated with a higher prevalence of chronic diseases. Males had a lower rate than females. The other minority respondents had lower rates than the Han majority and the Bai ethnic group. The illiterate respondents had the highest rate, and the middle school group had the lowest rate. The unemployed respondents had the highest rate, while students had the lowest rate. The major chronic diseases were hypertension (3.35%), rheumatism (1.92%), hyperosteogeny (0.53%), diabetes (0.45%), and gastropathy (0.38%).

The required hospitalization rate was 9.2%. The respondents aged 13–20 years had the lowest rate. For other age groups, the hospitalization rate increased with age. The divorced or widowed respondents had the highest rate, and the single respondents had the lowest rate. The very-low-income respondents had a higher rate than other income respondents. Males had a lower rate than females. The Bai ethnic group had a higher rate than the Han. The illiterates had the highest rate, and the high school and above group had the lowest rate. The unemployed respondents had the highest rate. The significant diseases requiring hospitalization were injury, hypertension, childbirth, heart disease, and appendicitis.

After adjusting the age proportion of the sixth population census of Yunnan Province, the 2-week prevalence was 7.1%, the prevalence of chronic disease was 10.7%, and the hospitalization rate was 8.4%. The gender composition was the same as that of the population census, which did not need to be adjusted.

There were linear trends between income and the prevalence of chronic diseases (χ^2^ = 187.110, *P* < 0.001), income and the required hospitalization rate (χ^2^ = 9.861, *P* = 0.002), education levels and the 2-week prevalence (χ^2^ = 35.549, *P* < 0.001), education levels and the prevalence of chronic diseases (χ^2^ = 381.834, *P* < 0.001), and education levels and the required hospitalization rate (χ^2^ = 73.840, *P* < 0.001). The prevalence of chronic diseases and the required hospitalization rate decreased when income increased. The 2-week prevalence, the prevalence of chronic diseases, and the required hospitalization rate decreased when the education level increased.

### Health Equity

**Figure 1:**
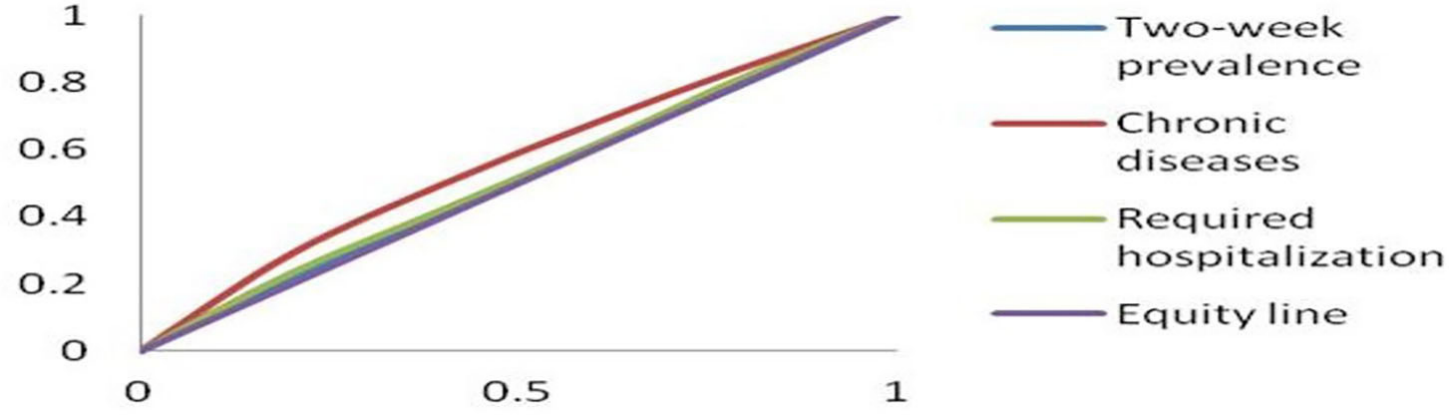
Concentration curves of health with different incomes.

**Figure 2:**
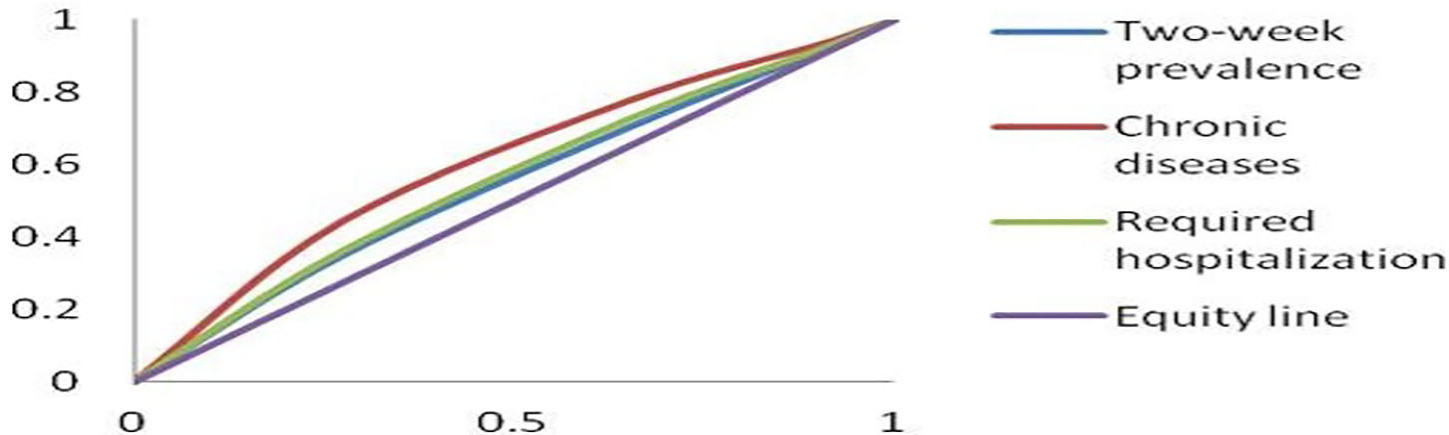
Concentration curves of health with different educational levels.

The CIs of the 2-week prevalence, the prevalence of chronic diseases, and the required hospitalization rate among residents with different incomes were −0.01888, −0.12520, and −0.03470, respectively (Figure 1). The CIs of the 2-week prevalence, the prevalence of chronic diseases, and the required hospitalization rate among respondents with different educational levels were −0.08296, −0.19424, and −0.10274, respectively (Figure 2).

### Factors Influencing Health

Multivariate logistic regression was used to analyze the 2-week prevalence, chronic diseases, and required hospitalization factors.

Table 2 shows the results of multivariate logistic regression of the 2-week prevalence. Gender, age, nationality, occupation, and the number of family members influenced the 2-week prevalence when other covariates were controlled. The 2-week prevalence for females was 1.19 times that of males. The prevalence of respondents aged 0–12, 13–20, 21–40 years were 1.54 times, 0.58 times, 0.60 times that of those aged 61 years and above, respectively. The 2-week prevalence for the Bai ethnic group was 1.27 times that of the Han respondents. The 2-week prevalence of workers was 0.43 times that of farmers. As the number of family members increased by one unit, the 2-week prevalence dropped by 11%.

**Table 2:** Logistic regression model fitting results.

**Dependent variables**	**Two-week prevalence**		**Chronic diseases**		**Required hospitalization**	
	**OR**	**95% CI for OR**	**OR**	**95% CI for OR**	**OR**	**95% CI for OR**
Number of family members	0.89*	(0.86, 0.92)	0.90*	(0.87, 0.92)	0.94*	(0.91, 0.96)
Female	1.19*	(1.08, 1.30)	1.39*	(1.28, 1.51)	1.25*	(1.14, 1.36)
Male (Reference)						
**Age (years)**						
0–12	1.54*	(1.14, 2.08)	0.05*	(0.03, 0.08)	0.57*	(0.42, 0.77)
13–20	0.58*	(0.42, 0.79)	0.06*	(0.04, 0.09)	0.37*	(0.27, 0.49)
21–40	0.60*	(0.51, 0.71)	0.15*	(0.13, 0.17)	0.37*	(0.33, 0.43)
41–60	1.02	(0.88, 1.18)	0.55*	(0.50, 0.61)	0.59*	(0.52, 0.66)
61+ (Reference)						
**Ethnicity**						
Bai	1.27*	(1.11, 1.47)	1.06	(0.93, 1.20)	1.38*	(1.22, 1.57)
Others	1.12	(0.89, 1.42)	1.00	(0.81, 1.25)	1.35*	(1.09, 1.66)
Han (Reference)						
**Occupation**						
Students	0.87	(0.69, 1.08)	0.81	(0.55, 1.19)	0.59*	(0.47, 0.75)
Workers	0.43*	(0.30, 0.60)	0.67*	(0.52, 0.86)	0.58*	(0.45, 0.76)
Unemployed	0.77	(0.57, 1.06)	1.14	(0.91, 1.42)	1.09	(0.85, 1.38)
Other	0.80*	(0.63, 1.01)	0.97	(0.80, 1.18)	0.86	(0.70, 1.06)
Farmers (Reference)						
**Marriage**						
Single	0.79	(0.58, 1.06)	0.45*	(0.34, 0.60)	0.58*	(0.45, 0.75)
Married	1.04	(0.85, 1.28)	0.93	(0.81, 1.06)	0.92	(0.78, 1.08)
Divorced or widowed (Reference)						
**Family Income per person (RMB per year)**						
<2400	1.03	(0.89, 1.20)	1.58*	(1.38, 1.79)	1.15	(1.00, 1.33)
2,400–4,399	1.00	(0.86,1.16)	1.28*	(1.12, 1.47)	1.05	(0.91, 1.21)
4,400–6,699	1.02	(0.88, 1.19)	1.25*	(1.09, 1.43)	1.13	(0.98, 1.30)
6,700–10,199	0.92	(0.79, 1.07)	1.11	(0.97, 1.27)	1.19	(1.03, 1.37)
10,200+ (Reference)						
**Education**						
Illiterate	1.04	(0.82, 1.33)	1.66*	(1.32, 2.08)	1.25	(0.98, 1.59)
Primary school	0.90	(0.70, 1.14)	1.44*	(1.15, 1.81)	1.25	(0.98, 1.58)
Middle school	0.91	(0.71, 1.16)	1.20	(0.95, 1.52)	1.11	(0.87, 1.42)
High school	0.87	(0.63, 1.19)	1.28	(0.96, 1.72)	1.20	(0.89, 1.63)
Above high school (Reference)						

In logistic regression, we used the ENTER method. All the independent variables have been included in the analysis, and the corresponding OR value of each variable is obtained by adjusting the influence of other independent variables. *P < 0.05.

The factors influencing the prevalence of chronic diseases were gender, age, occupation, marriage status, income, education level, and the number of family members, when other covariates were controlled. The prevalence of chronic diseases for females was 1.39 times that of males. The prevalences for the respondents aged 0–12, 13–20, 21–40, 41–60 years was 0.05, 0.06, 0.15, and 0.55 times that of those aged 61 years and above, respectively. The prevalence for workers was 0.67 times that of farmers. The prevalence for single respondents was 0.45 times that of divorced or widowed respondents. For respondents with very-low, low-, middle-income, the prevalences were 1.58, 1.28, and 1.25 times that of very-high-income, respectively. The prevalences for the illiterates and the primary school groups were 1.66 and 1.44 times those of the high school above group, respectively. The prevalence of chronic diseases decreased by 11% as the number of family members increased by one unit.

The factors influencing the required hospitalization rate were gender, age, nationality, occupation, marriage status, and the number of family members, when other covariates were controlled. The required hospitalization rate for females was 1.25 times as males. The required hospitalization rate for the respondents aged 0–12, 13–20, 21–40, 41–60 years were 0.57, 0.37, 0.37, and 0.59 times that of those aged 60 years and above, respectively. The required hospitalization rate for the Bai respondents and other ethnic respondents were 1.380 times and 1.348 times as the Han respondents, respectively. The required hospitalization rate for students and workers were 0.59 times and 0.58 times that of farmers, respectively. The required hospitalization rate for single respondents was 0.58 times that of divorced or widowed respondents. With each unit increased in the number of family members, the required hospitalization rate decreased by 6.5%.

## Discussion

Compared with the report of the 5th NHSS in China, we found that the 2-week prevalences and the prevalences of chronic diseases among the sampled residents were lower (7.3 vs. 24.1%, 12.8 vs. 33.1%, respectively). However, this result was similar to those of other studies in Yunnan (13, 14). The required hospitalization rate was similar to the 5th NHSS (9.2 vs. 9.0%). The possible reasons for the discrepancies are differences in the areas and ages of the samples. According to the 5th NHSS, the prevalence of disease among urban residents was higher than rural residents. The respondents in our study were sampled from rural area, while the respondents in the 5th NHSS included urban and rural areas. The respondents in our study included residents of all ages, whereas the respondents in the 5th NHSS were aged 15 and above.

There were income-related inequities in health. The CIs reflecting the health equity among different incomes were negative, suggesting that the respondents with lower income required more health resources than those with higher income and were less healthy, which agree with those of previous studies (15, 16). Van Doorslaer et al. (17) found that health inequalities benefited high-income individuals in nine countries. Van Doorslaer and Koolman (18) found that significant health inequalities were beneficial to high-income individuals in 13 European countries. These studies also support our results of income-related health inequalities.

Social position exerts a powerful influence on the type, magnitude, and distribution of health in high-, low- and middle-income counties (19). Education, income, and occupation are critical factors in determining social status and gaining power and social resources. Compared with the high-income group, people with lower income are more likely to experience financial stress, and economic difficulty was a significant obstacle to health care access. Income was also related to lifestyle; those with low income were more likely to smoke, drink excessively, and be overweight and inactive (20). Unhealthy lifestyles, financial stress, and lower health service utilization levels lead to less health in low-income people. Our study also demonstrated that the CI of the prevalence of chronic diseases (−0.12520) was larger than that of the 2-week prevalence (−0.01888) and the required hospitalization rate (−0.03470) among the respondents with different incomes, suggesting that income had a more significant impact on equity of chronic diseases than on required hospitalization or 2-week prevalence. The linear trend tests results also indicated linear trends existed between income and the prevalence of chronic diseases and between income and the required hospitalization rate.

The CIs reflecting health equity among educational levels were negative, suggesting that respondents with lower educational levels required more health resources, and had worse health status than residents with higher educational levels (21). People with higher education levels have more knowledge about health and health care and have better self-management and healthier lifestyles, and can better utilize health care services. A healthy lifestyle can help people prevent and control diseases, such as chronic diseases and weight gain. Timely diagnosis and treatment can prevent mild illness from becoming severe (22–24). In our study, the CIs of the prevalence of chronic diseases (−0.19424) and the required hospitalization rate (−0.10274) were larger than that of the 2-week prevalence (−0.08296) among the respondents with different educational levels, suggesting that lower education level is a primary factor influencing inequity of chronic diseases and hospitalization, and people with lower educational levels had poorer health knowledge and health care consciousness. Educational level positively correlated with income and age, as low educational level correlated with low income and old age. The older and low-income residents had higher chronic disease rates and hospitalization rates compared to their counterparts. More resources should be allocated to rural populations with low income and low educational levels, and health knowledge should be disseminated in a simple, visual, and easily understandable way, which is conducive to disease prevention and control, especially for chronic diseases.

The females had higher required hospitalization rates than males. Women had more risk of diseases because of their particular physiological structure, including maternity and gynecological diseases (25–27). The 5th NHSS in China showed that the 2-week prevalence and the prevalence of chronic diseases in females were higher than in males. Ren's study (26) found that females had a higher prevalence of chronic diseases than did males; however, Cheng et al. (28) showed the opposite result. This discrepancy might be caused by regional differences, as Cheng's study was in a developed area (Minhang District of Shanghai). Ours and Ren's study were in the countryside of under-developed areas (Yunnan and Ningxia Province). Compared to men, women had lower mortality, but they tended to be sicker than men and have more significant morbidity, worse health-related quality of life, and worse perception of health, including higher levels of depression, psychiatric disorders, and various chronic illnesses (29, 30). In China's rural areas, men are dominant in the family and society, and most resources were allocated to them; there are differences between males and females in education, employment, and economic empowerment (31, 32). It is more likely that women have less power, lower-income, and long-term housework, the health status of other family members, especially male family members, takes precedence over women's health (33). In rural areas, heavy physical labor, economic dependence on men, and lower status in a family all adversely affect women's health.

Compared to farmers, workers required fewer health resources, and students had lower required hospitalization rates. Farmers are less educated than workers, lack healthcare knowledge, and engage in challenging physical work for long periods, leading to poor health status and high morbidity. Chronic disease is an essential factor influencing the required hospitalization rate. Students are young and rarely have chronic diseases. They are the focus of family and health systems, so they have good healthcare, resulting in a low required hospitalization rate.

Ethnicity was a factor influencing the 2-week prevalence and the required hospitalization rate. Compared to the Han respondents, the Bai respondents had a higher 2-week prevalence and a higher required hospitalization rate; other ethnic respondents had higher required hospitalization rates. Previous studies (27, 34, 35) showed that ethnic minorities had worse health status than Han people. The minority respondents had low educational levels and particular lifestyle and eating habits, including drinking problems. When they became ill, they sought the help of non-professional medical persons or medicine men in their villages. This led to poor health conditions.

Marriage status was also a factor influencing rates of chronic diseases and required hospitalization. The divorced or widowed had higher prevalences of chronic diseases and required hospitalization rates than the singles. Education level, marriage status, and age were closely related. The divorced or widowed were relatively older. Older age correlated with lower education level and worse health condition (35).

However, one limitation of our study is that the effect of family clustering was not considered in the data analysis, that is, we have not considered residents in the same household share some similarities and are not independent sampled. This should be discussed in our further study.

## Conclusions

Age, gender, income, education level, and marital status were factors influencing health conditions. More attention should be paid to the aged, females, residents with low income and low educational levels, the divorced or widowed, and residents with chronic diseases. Providing more health knowledge, especially the (35) prevention and treatment of chronic diseases, to residents with lower education levels or older ages will help them carry out health promotion activities and improve their self-management ability and health levels.

There are health inequities among Yunnan residents, especially concerning chronic diseases and the required hospitalization across income and education levels. The respondents with low income and low education levels had worse health status.

## Data Availability Statement

The original contributions presented in the study are included in the article/supplementary material, further inquiries can be directed to the corresponding author/s.

## Ethics Statement

The studies involving human participants were reviewed and approved by the Ethics Committee of Kunming Medical University. Written informed consent to participate in this study was provided by the participants' legal guardian/next of kin.

## Author Contributions

X-ML, L-PH, ZY, and QM developed the idea and contributed to the study design. X-ML and L-PH carried out the analysis. L-PH wrote the manuscript and takes responsibility for the overall content of the paper. JK and Y-YX revised the manuscript. X-ML, JK, ZY, Y-YX, and QM commented on the paper and have seen and accepted the final version. All authors contributed to the article and approved the submitted version.

## Conflict of Interest

The authors declare that the research was conducted in the absence of any commercial or financial relationships that could be construed as a potential conflict of interest.
